# Bibliographical

**Published:** 1887-04

**Authors:** 


### BIBLIOGRAPHICAL.

THE MICROSCOPIC STRUCTURE OF A HUMAN TOOTH, TOGETHER WITH SOME UNUSUAL AND IRREGULAR FORMS OF TEETH. By C. H. STOWELL, M. D., F. R. M. S., Professor of Histology and Microscopy in the University of Michigan. Chas. H. Stowell, Publisher, Ann Arbor, Mich. Price, $6.00.

This work is issued in the form of an atlas, with a very beautiful leather case or cover for holding and protecting it. The engravings, many of which are full-page, are beautifully done, and the letter-press is like that of some holiday annual. The full-page plates are: a molar; a longitudinal section of an incisor; a longitudinal section of a lower molar; a transverse section of a bicuspid near the bifurcation of the roots; the blood-vessels of the pulp; a section of the root of an incisor parallel to the dentinal tubuli; two greatly enlarged dentinal fibrils; odontoblasts; sections of enamel, etc. Each plate is accompanied by an explanatory text, and there are directions for the preparation of microscopical sections of the teeth, cuts of dental anomalies, etc., etc.

Prof. Stowell is known as a very competent microscopist, formerly the editor of *The Microscope,* a monthly journal devoted to microscopical science, and as the author of a number of works on pathological subjects. He is a clear and forcible writer, and a careful and painstaking observer. Nevertheless, he will find those who will take issue with him on some of his theories. The dentinal fibrils he represents as direct offshoots from the odontoblast cells. This is not the testimony of some other competent observers. There are other criticisms which might be made, and yet, despite these imperfections, the work is the handsomest one of the kind that has been issued from the American press. We have had it in use in college lectures, and have found it a great convenience. The delicacy of the drawing and the clearness of the engraving make it the most useful work of the kind for those engaged in teaching with which we are acquainted.

C. W. Arnold, Detroit, is the general agent for the United States. Sold by subscription only.

FIELD'S MEDICO-LEGAL GUIDE; for Doctors and Lawyers. By GEO. W. FIELD, LL. B. Albany and New York: Banks & Brothers. 1887.

This is a book which treats of the legal liability of physicians in practice, and of their duties under the law. It embraces the following subjects: Medical Witnesses; Medical Expert Testimony; Insanity and its Legal Relations; Privileged Communications; Abortion; Civil Liability of Medical Men for Malpractice; Criminal Liability for Malpractice; Liability for Practicing in Violation of Statutes; Damages; Compensation; Medical Ethics. In fact, it is a text-book of medical jurisprudence. What between jealous practitioners of medicine and conscienceless practitioners of law, both of whom have a direct interest in fomenting misunderstandings between patient and doctor, it behooves the latter well to know the ground upon which he stands, and to comprehend what are his legal rights and liabilities. Of these this book will thoroughly inform him, and it is not too much then to say that it should be in the hands of every practitioner of the healing art. It has a copious index and a very complete table of cases and decisions, so that it is of value to the lawyer as well as the medical man.

Transactions of the American Dental Association at the Twenty-sixth Annual Session, held at Niagara Falls, N. Y., commencing Aug. 3, 1886. Philadelphia: The S. S. White Dental Manufacturing Co.

It is unnecessary for us to refer particularly to the contents of this annual volume, inasmuch as a full report of the proceedings was published in this journal immediately subsequent to the meeting. It makes a volume a little larger than that of 1885, and quite equal to it in appearance. The publications of the S. S. White Dental Manufacturing Co. are always neat in their typography and binding.

Wear and Tear; or Hints for the Overworked. By S. Weir Mitchell, M. D., LL. D. Fifth edition. Thoroughly revised. Philadelphia: J. P. Lippincott Company. 1887.

Prof. Mitchell is widely known as one of the most competent authorities upon neural diseases that the medical profession of America has in its ranks. Fifteen years ago he issued this little work "as a warning to a restless nation possessed of an energy tempted to its largest uses by unsurpassed opportunities." Since that day hygienic matters have been more carefully observed, and there have been constant improvements in national dress, diet and education, followed by a marked physical change for the better in our development, until the lank, lean and sallow Yankee of a generation ago, is no longer typical of American physique. To this altered development the writings of Dr. Mitchell have largely contributed, and this book is one of his best.

An Epitome of the Newer Materia Medica. To which is added a property and dose list. Fourth edition. Revised and enlarged. Detroit: Parke, Davis & Company. 1886.

This is a classified list of the properties and qualities of the standard medicinal specialties introduced and manufactured by those reliable pharmacists, Parke, Davis & Co., of Detroit. This firm has succeeded in establishing a reputation for skill in manufacturing and uprightness in dealing, which has made their preparations standard the world around. They prepare many compounds for dentists, and, so far as we know, are the only manufacturing chemists who have taken particular pains to prepare pure drugs especially for their use.

Transactions of the California State Dental Association, at the 13th, 14th, 15th, 16th and 17th Annual Sessions, held in San Francisco.

This makes a voluminous work of over 500 pages, containing some valuable papers and discussions. But it also includes considerable matter which should never have seen the light. It is not edifying to read of the personal quarrels and altercations which sometimes are allowed to take place in society meetings. When dirty linen is to be washed it cannot be done too privately, and we can but wonder that the publication committee should have published to the world the record of the quarrels which resulted in a divided profession in the State of California.

Transactions of the Dental Society of the State of New York. Eighteenth Annual Meeting, held at Albany, May, 1886.

This is not a very voluminous report, as it covers but about fifty pages, a considerable portion of these being occupied by reports of routine business, district societies, etc. What there is, however, is very well edited and printed, but the Dental Society of the great State of New York should make a better showing.

The Management of Pulpless Teeth.

This is a capital little pamphlet, published by the Odontological Society of Chicago as a hand-book of reference for dental practitioners. It contains the essentials of pulp and root treatment in a condensed form, and although there will be a difference of opinion concerning certain methods, its perusal will be beneficial to any practitioner.

*Scavi di Capodimonte: Sopra un Ritratto di Gneo Pompeo Magno.* W. Helbig. Roma.

*Illustrated Catalogue of the Bausch & Lomb Optical Co.* Rochester, N. Y., and New York City.

*President's Address,* Tenth Annual Meeting of the Detroit Medical and Library Association. By C. J. Lundy, A. M., M. D.

*The Doctorate Address.* Delivered at the Semi-Centennial Anniversary of the University of Louisville, March 2, 1887. By David W. Yandell, M. D.

*Cases in Orthopædic Surgery,* By Ap. Morgan Vance, M. D. Reprinted from *The New York Medical Journal.*

*Presidential Address.* Delivered before the Louisiana State Dental Society. By Geo. J. Friedrichs, M. D., D. D. S.

*Courtesy Among Dentists at Professional Meetings.* Annual oration delivered before the American Academy of Dental Science, Boston, Nov. 10, 1886. By J. N. Farrar, M. D., D. D. S.

*On Certain Mooted Points in Gynecology.* By Thomas Addis Emmet, M. D. Reprinted from *The British Medical Journal.*

*Researches into the Etiology of Dengue.* By J. W. McLaughlin, M. D. Reprinted from the Journal of the American Medical Association.

*Follicular Amygdalitis.* By A. Jacobi, M. D. Reprinted from *The Medical Record.*

*Is Life-force Matter?* By J. Hardman, D. D. S. Address before the Muscatine (Ia.) Academy of Science.

*Second Annual Report of the Minnesota State Board of Dental Examiners.*

*Hyperostosis of Roots of Teeth.* By Frank Abbott, M. D. Read before the American Dental Association at Niagara Falls. Reprinted from the *Dental Cosmos.*

*Some Notes of Recalcification of Human Teeth.* Read before Sections F and H in joint session at the Buffalo meeting of the A. A. A. S., August, 1886. By J. R. Walker, D. D. S.

